# Bibliometric analysis on CRISPR/Cas: a potential Sherlock Holmes for disease detection

**DOI:** 10.3389/fmolb.2024.1383268

**Published:** 2024-07-11

**Authors:** Rohan Samir Kumar Sachan, Adarsh Choudhary, Inderpal Devgon, Arun Karnwal, Abdel Rahman Mohammad Said Al-Tawaha, Tabarak Malik

**Affiliations:** ^1^ School of Bioengineering and Biosciences, Lovely Professional University, Phagwara, India; ^2^ Department of Biological Sciences, Al Hussein Bin Talal University, Ma’an, Jordan; ^3^ Department of Biomedical Sciences, Jimma University, Jimma, Ethiopia

**Keywords:** clinical studies, bibliometrics, database, CRISPR, disease, detection

## Abstract

CRISPR has revolutionized illness detection by using precision gene editing to identify specific sequences in recent years. Using the Scopus database, this study performs a comprehensive bibliometric analysis, looking at academic papers on CRISPR that were published between 1992 and 2023. After screening a dataset of 1407 articles using Zotero, trends in annual publishing, citation patterns, author affiliations, and keyword co-occurrence are revealed using analysis tools such as VOSviewer, RStudio, and MS Excel. According to the report, there was only one CRISPR publication in 1992. By 2017, there were a meager 64 papers. Nonetheless, there is a notable upsurge between 2018 and 2023. Leading nations involved in CRISPR-based illness detection research include Germany, the United States, China, India, and the United Kingdom. Chongqing University Three Gorges Hospital, Chongqing University Medical University, and Chongqing University Bioengineering College are a few of the top institutions. With the greatest publication numbers (1688 and 1616) and strong total link strengths (TLS) of 42 and 77, respectively, authors Liu, C., and Li, Y., stand out. The field with the greatest citation counts as of 2023 is Broughton’s 2020 study on CRISPR-based SARS-CoV-2 detection in Nature Biotechnology, with 1598 citations. Biosensors and Bioelectronics comprise 14.99% of papers. Researchers, decision-makers, and interested parties can use this thorough summary to help them make well-informed decisions about future CRISPR-based disease detection studies.

## Introduction

Since its discovery in 1987, the CRISPR-Cas system, a groundbreaking tool in genomic manipulation, has undergone substantial evolution (Nidhi et al., 2021). Initially developed for genetic and infectious disease treatments. The applications of CRISPR-Cas have since broadened to encompass a wide range of fields, including molecular visualization (Batool et al., 2021; Wang et al., 2023). In 2016, there was a significant milestone when CRISPR-Cas was successfully integrated into molecular diagnostics, resulting in a fundamental change in illness diagnosis (Weng et al., 2023). The next few years saw the rise of CRISPR-Cas diagnostic tools, which were notable for their high sensitivity, specificity, cost-effectiveness, and decreased dependence on sophisticated technology (Mathews et al., 2015).

A bibliometric analysis is proposed to examine scholarly articles linked to CRISPR that were published between 1992 and 2023, in response to the increasing demand for CRISPR-based research, particularly following its recognition with the Nobel Prize. Taking cues from Sherlock Holmes, this analysis seeks to decipher the complexities of CRISPR/Cas technology, serving as a metaphorical investigation instrument (Baik et al., 2022). The study aims to gain a thorough knowledge of the changing landscape of CRISPR research in disease detection by analyzing yearly publishing patterns, citation trends, author affiliations, and keyword co-occurrence. This metaphorical instrument is positioned to illuminate the interrelationships and significance of research publications within this ever-changing domain.

The bibliometric study utilizes a rigorous technique, making use of the Scopus database and software tools such as VOS Viewer, RStudio, and MS Excel (Ejaz et al., 2022; Khurshid Wani et al., 2023). The study utilizes a dataset consisting of 1407 publications to analyze and identify prominent authors, collaborative networks, and topic clusters that contribute to the discussion on CRISPR/Cas as a powerful diagnostic tool. The analysis provides useful insights into the current level of research and is a crucial resource for researchers, policymakers, and stakeholders interested in CRISPR-based disease detection research. It helps in making informed decisions for future endeavors.

## Material and Methodology

### Methodology and approach to research design and information retrieval

**FIGURE 1:**
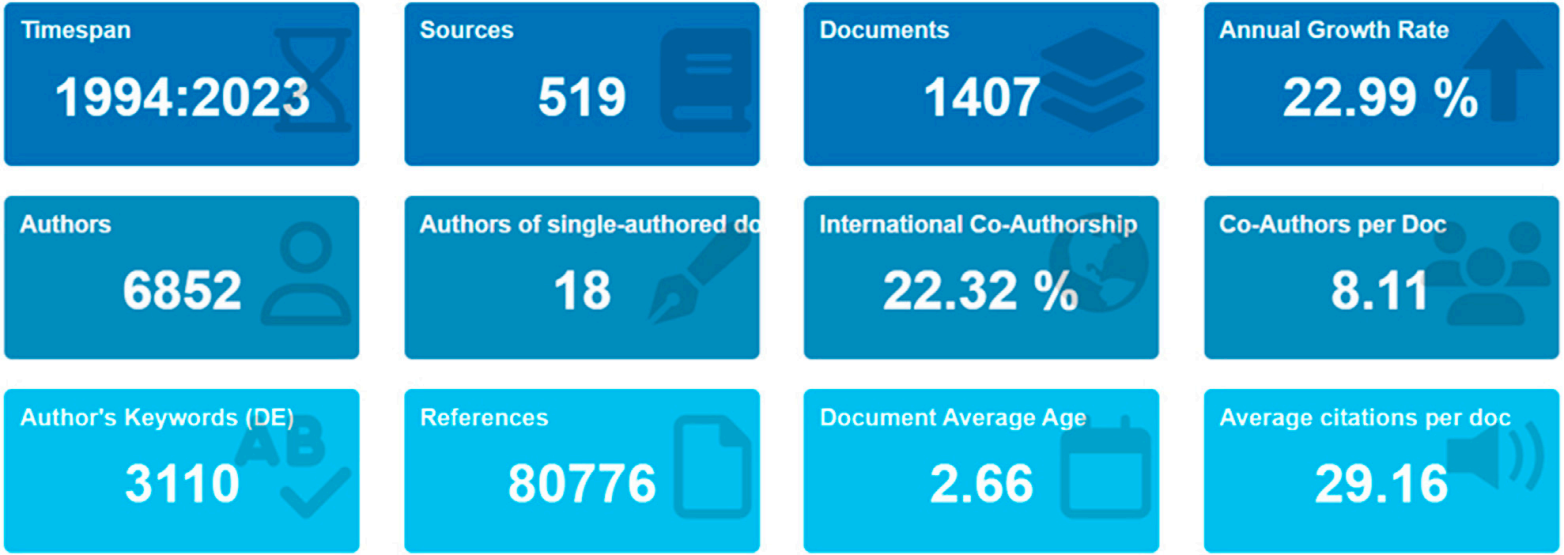
Information on the data generated by the SCOPUS databases for CRISPR-based disease detection.

The selection of techniques and tools is essential in the process of data analysis during research. Google Scholar, Scopus, and Web of Science (WoS) are highly prominent and extensively used databases for accessing scientific information from publications (Pranckutė, 2021). We conducted an extensive bibliometric analysis to examine literature on the identification of illnesses using CRISPR technology. The examination specifically targeted academic articles published in publications indexed in the SCOPUS databases between 1994 and 2023. We used the SCOPUS indexing system to find relevant publications and extracted the bibliographic information of these articles using the database’s export tools. The Scopus database offers extensive access to important scholarly materials that guarantee the production of high-caliber scientific papers (Abdullah Sani et al., 2023). We obtained the data for CRISPR-based disease detection by performing a search query on 3 January 2024, in the Scopus database using the search term TITLE-ABS-KEY (“CRISPR” AND disease AND detection). A total of 1589 publications were acquired, specifically articles and reviews as document types and journals as source types. In addition, the language and publication stage was restricted to English, resulting in a total of 1407 publications after applying various limits (Figure 1). We exported the acquired records in “csv” format to facilitate subsequent bibliometric analysis. The data was thoroughly examined to analyze and eliminate any duplicate entries using Zotero (v.6.0.7). However, the analysis did not detect any duplicates, and the data was deemed suitable for further analysis.

### Data analysis and visualization

The requirement for a comprehensive and cooperative analysis of literature has increased significantly in parallel with notable breakthroughs. Bibliometric approaches have become an essential tool for systematically analyzing bibliographic databases, providing a quantitative approach for this purpose (Linnenluecke et al., 2020). This strategy classifies and arranges scientific material according to variables such as journals, authors, institutions, and countries. This strategy also enhances researchers’ comprehension of study domains. Our study on CRISPR-based illness identification involved a thorough statistical analysis employing bibliometric analysis. We did a comprehensive study on articles obtained from the Scopus database, utilizing a wide range of analytical methods, including citation and publication analysis, author and co-authorship analysis, co-occurrence and keyword analysis, as well as network and mapping research. For the analysis of these analyses, a range of tools and software applications were employed, such as VOSviewer (version 1.6.18), RStudio (version 2022.02.2), and MS Excel. The scientific map study was examined using Biblioshiny, a freely available software incorporated within RStudio’s bibliometrix package. Ultimately, the Biblioshiny software was employed to produce a three-field plot analysis, which visually depicts the relationships among keywords, sources, and nations.

## Result and discussion

### Yearly distribution and amount of published works

**FIGURE 2:**
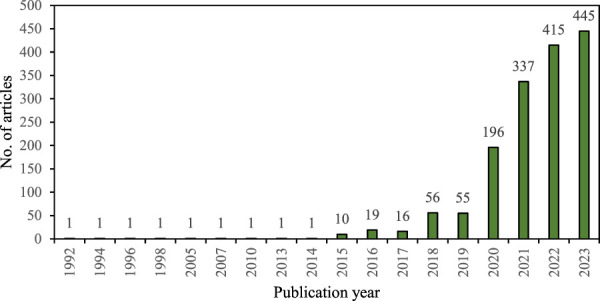
Annual distribution and publication trends of CRISPR/Cas system in disease detection.

Evaluating the trend of increased publication of CRISPR-based illness detection can offer useful insights into prospective future research fields and solutions to overcome obstacles in medical diagnostics (Ibrahim et al., 2020). The number of publications showed steady growth from 1992 to 2023, as seen in Figure 2. The inaugural article on CRISPR was published in 1992 and remained consistent until 2014. There was a modest rise in publications from 2015 to 2017. From 2017 to 2023, there was a significant and rapid growth in the total number of published works. In 2023, there were 45 publications, followed by 415 in 2022 and 337 in 2021. Notably, the period from 2020 to 2023 accounted for almost 88.8% of the publications. However, the publication increases over a span of 30 years proved to be unpredictable. Barrangou and Doundna’s (2016) (Barrangou and Doudna, 2016) study found a rise in the number of publications between 2012 and 2015 in the Web of Science database, aligning with the observed pattern. In addition, Wang et al. (2020) emphasized in their comprehensive analysis of CRISPR-Cas articles in the Web of Science that there has been a noticeable rise in the number of publications starting in 2015 and 2016 (Wang et al., 2020).

Based on the data provided, it’s evident that there has been a significant increase in CRISPR-related publications during the COVID-19 pandemic compared to the pre-pandemic period. From 1992 to 2019, there were a total of 165 CRISPR-related articles, whereas from 2020 to 2023, there were 1393 articles, indicating a substantial surge in research activity during the pandemic years. Furthermore, among the 1393 articles published during the pandemic period, 446 were specifically related to COVID-19, demonstrating the heightened interest in applying CRISPR technology to address challenges posed by the pandemic. The substantial surge in publications, particularly those related to COVID-19, underscores the significant impact of the pandemic on driving research efforts toward leveraging CRISPR technology to address challenges posed by the virus. The increase in COVID-19-related CRISPR articles also suggests a heightened technological hype surrounding CRISPR during the pandemic, as researchers sought innovative solutions to combat the virus. Furthermore, the presence of 446 COVID-19-related CRISPR articles indicates substantial efforts towards the translation and application of CRISPR for antiviral therapeutics, diagnostics, and vaccine development.

### Comprehensive analysis of CRISPR-Cas applications in disease detection, sample sources, and protein utilization

In our investigation of diseases and their detection via CRISPR-Cas systems, we uncover a rich landscape of research articles spanning various health conditions. Notably, COVID-19 emerges as a focal point, with 446 articles dedicated to leveraging CRISPR-Cas systems for rapid and sensitive detection of the SARS-CoV-2 virus. This underscores the urgent need for effective diagnostic tools during the ongoing pandemic. Additionally, tuberculosis (TB) detection garners attention, with 11 articles illustrating the potential of CRISPR-Cas systems in revolutionizing TB diagnosis, particularly in resource-limited settings. Moreover, a significant number of articles focus on detecting infectious diseases such as HIV, HPV, MRSA, *Neisseria gonorrhoeae*, malaria, and *Listeria*, showcasing the versatility of CRISPR-Cas systems in combating diverse pathogens. Furthermore, exploration into CRISPR-Cas technology for cancer detection demonstrates promising avenues for early diagnosis and personalized treatment strategies.

Our analysis extends beyond disease focus to encompass sample sources utilized in CRISPR-based diagnostic methodologies. Saliva emerges prominently, with seven articles showcasing growing interest in non-invasive diagnostic approaches. Food samples feature prominently as well, with 14 articles emphasizing the importance of CRISPR-based methods in ensuring food safety. Detection of pathogens in environmental samples like wastewater and sewage water underscores CRISPR-Cas systems’ potential in environmental surveillance. Moreover, the versatility of CRISPR-based diagnostics extends to blood and milk samples, indicating efficacy across diverse biological matrices.

Furthermore, our investigation unveils a diverse spectrum of CRISPR-associated proteins utilized across the literature. Cas12a emerges as the most prevalent, featured in 330 articles, followed by Cas9 (98 articles) and Cas13 (77 articles). Despite their dominance, alternative CRISPR-associated proteins such as Cas3, CasX, C2c1, and Cpf1 are also explored, albeit in smaller numbers. Their inclusion in the literature signifies ongoing exploration and diversification of CRISPR-based technologies, enriching our understanding of CRISPR-Cas system utilization and prevalence.

### Publication distribution by countries

**FIGURE 3:**
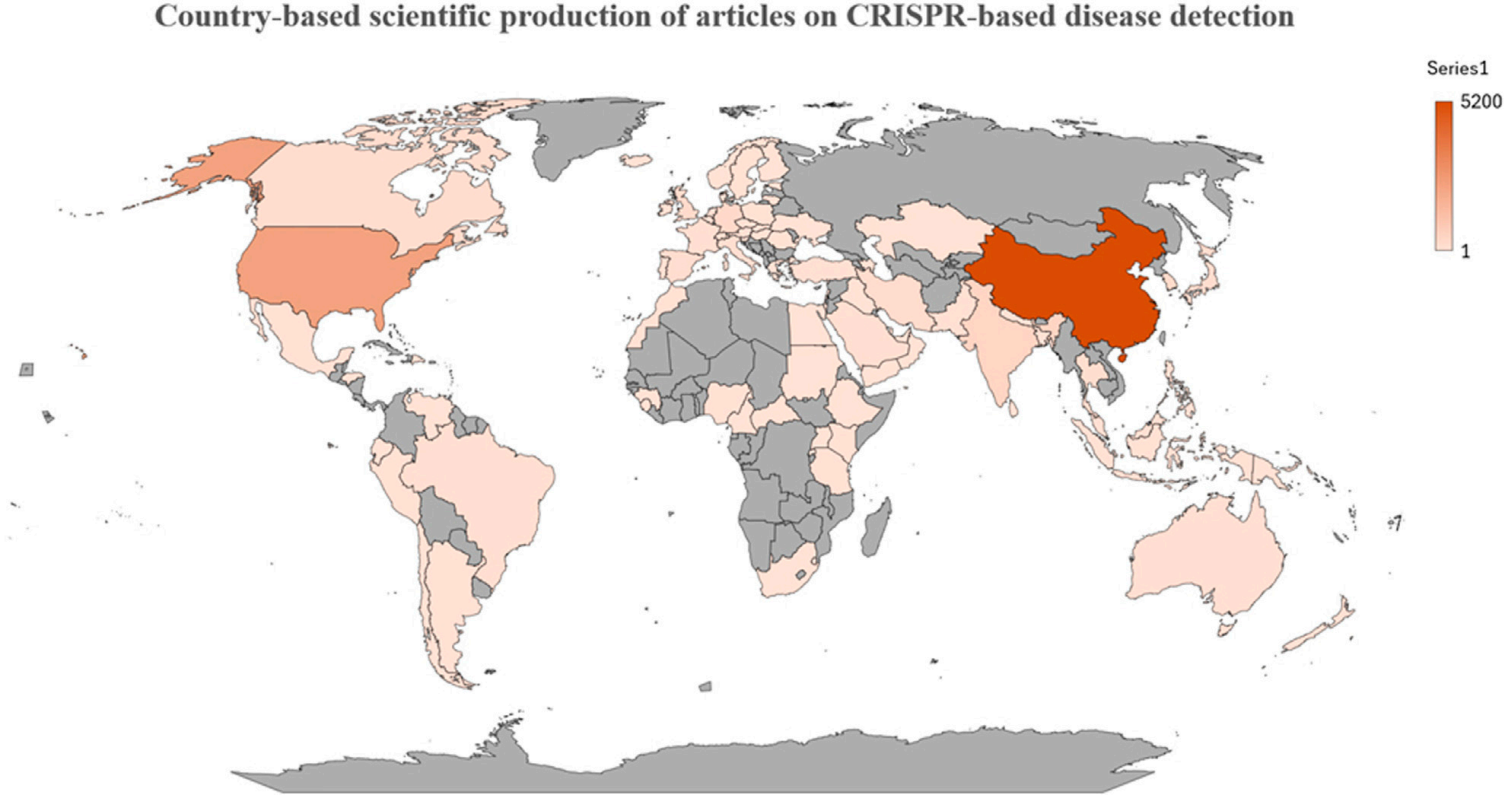
Publication distributions of disease detection using CRISPR technology throughout different countries.

**FIGURE 4:**
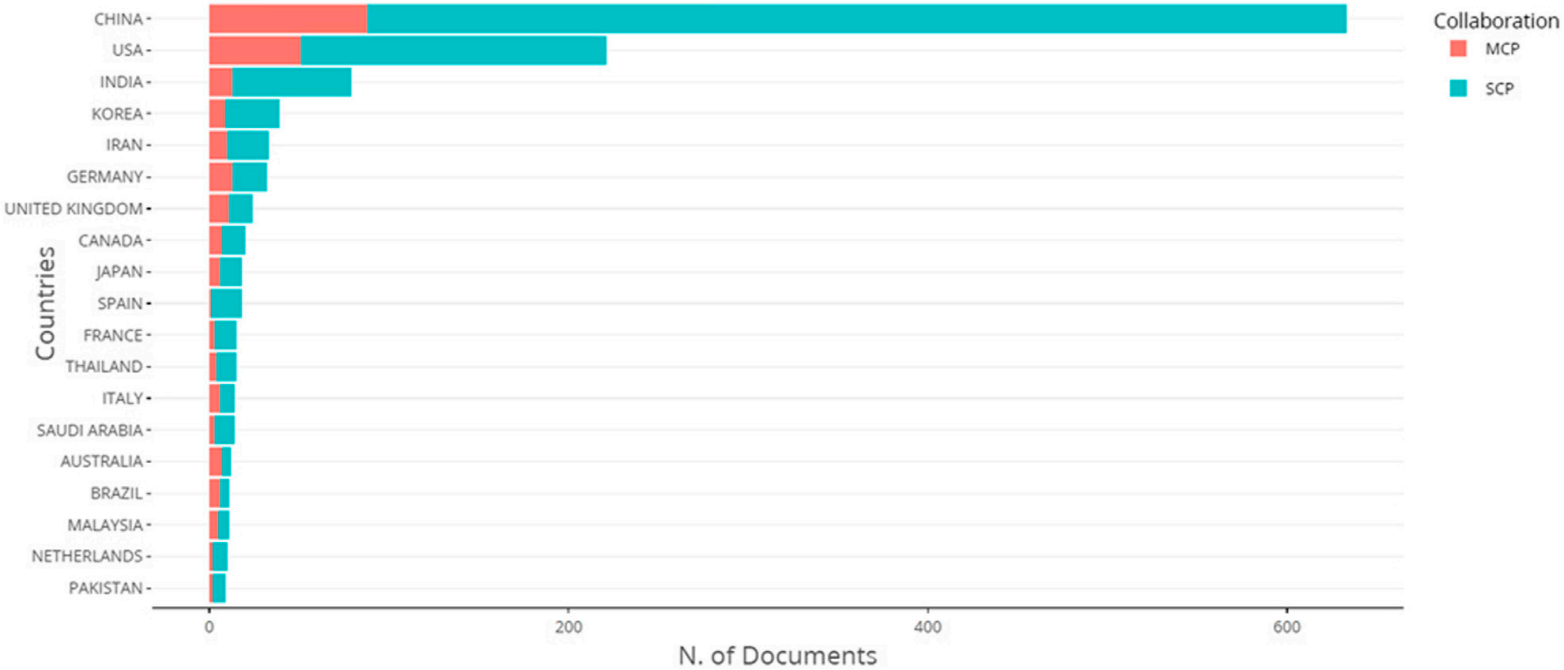
International and domestic cooperation facilitated by MCP and SCP indicators. The acronym MCP denotes international collaboration between multiple countries, whereas SCP refers to research generated solely by a single country. The nations chosen for analysis were determined depending on the nationality of the relevant author.

There were a total of 1772 research articles recorded in 30 countries. Supplementary Table S1 summarizes each country with the total number of publications in the field of CRISPR-based disease detection. China had the most publications with 690 (38.98%), the United States came in second with 19.41%, and India came in third with 5.30%. The United Nations, Germany, South Korea, Iran, and Canada are just a few of the organizations that have contributed to the body of research. Each of these contributors has published articles, with a range of 30–70 publications. The remaining countries contributed to the literature by collectively producing a total of papers equal to or greater than 5, as shown in Table 1 and Figure 3. These observations indicate that the production of published literature is primarily attributed to developed nations with significant financial resources. As a result, the main focus of the partnership was with these advanced countries. India, China, South Korea, and Iran have emerged as the leading Asian countries showing significant interest and involvement in CRISPR-Cas research. Similar to India, there has been a notable increase in CRISPR research activities in recent years. The Indian Council of Agricultural Research (ICAR), a well-regarded research institute, has the highest number of publications (22) focused on agricultural applications. The Council of Scientific and Industrial Research also saw an increase in publications, with 17 in the years 2019 and 2020 (Roy et al., 2021). In our study, we examined both individual country publications (SCPs) and publications involving multiple countries (MCPs) to investigate the various collaboration models used in publishing articles related to CRISPR research. This analysis is presented in Figure 4 and Supplementary Table S2. As of 2023, China has the biggest number of special category producers (SCPs) compared to major category producers (MCPs). India has a greater number of major contributing parties (MCPs) in CRISPR research compared to Korea and Iran, as indicated by Supplementary Figure S1. The evaluation of joint research efforts involving many countries was conducted using a specific measurement called Total Link Strength (TLS), as described in Table 1. TLS functions as a comprehensive indicator of the strength of links across multiple factors, including joint endeavors among different nations and the published output of our research study. This indicator is crucial for measuring the overall influence that can be observed in bibliographic data. The analysis of collaborative research projects using the TLS ratings shows that the United States has emerged as the primary contributor, receiving a significant score of 226 out of 344 publications. China obtained the second rank with a TLS score of 157, while the United Kingdom closely followed with a score of 93, claiming the third position. The examination of the Country Cooperation Network Map, together with TLS scores, highlights the importance of collaborative partnerships in CRISPR research, with significant participation from the United States, China, the United Kingdom, Germany, Canada, and Australia (Figure 4). According to the data, papers originating from the United States had the most number of citations, totalling 19322 (equivalent to an average of 56.16 citations per year). China follows with 12888 citations (an average of 157 per year), then the United Kingdom with 2149 citations (an average of 33.06 per year), and Germany with 1961 citations (an average of 32.14 per year). Nigeria ranks 26th in terms of the quantity of articles but excels in the performance of the average citation number per article, with a score of 61.22. There are a total of nine articles with a combined total of 551 citations. Denmark currently holds the greatest average citation per paper in the field of CRISPR-based illness detection, with the U.S. following closely at 56.16, Brazil at 55.50, and Austria at 53.62 (Table 1). For more detailed information about country total citations scores contributed to the CRISPR-based disease detection is listed in Supplementary Table S3.

**TABLE 1:** Countries having at least 5 published studies on CRISPR-based disease detection. The TLS provides a glimpse into the synergistic prowess across diverse nations.

S. No	Country	Documents	Percentage (%) of documents	Citations	Average citations	Total link strength
1	China	690	38.94	12888	18.67	157
2	United States	344	19.41	19322	56.16	226
3	India	94	5.30	1625	17.28	52
4	United Kingdom	65	3.67	2149	33.06	93
5	Germany	61	3.44	1961	32.14	64
6	South Korea	56	3.16	1403	25.05	51
7	Iran	44	2.48	1064	24.18	45
8	Canada	38	2.14	1357	35.70	59
9	Australia	30	1.69	840	28.00	58
10	Spain	28	1.58	569	20.32	42
11	France	28	1.58	783	27.96	29
12	Japan	27	1.52	669	24.77	23
13	Saudi Arabia	27	1.52	839	31.07	21
14	Italy	25	1.41	1305	52.20	54
15	Taiwan	21	1.19	299	14.23	16
16	Netherlands	19	1.07	713	37.52	24
17	Turkey	19	1.07	513	27.00	23
18	Brazil	19	1.07	1056	55.50	15
19	Thailand	18	1.02	588	32.66	17
20	Russian Federation	18	1.02	222	12.33	14
21	Switzerland	16	0.90	616	38.50	24
22	Malaysia	15	0.85	447	29.80	32
23	Sweden	13	0.73	290	22.30	20
24	Singapore	11	0.62	441	40.09	21
25	Bangladesh	10	0.56	215	21.50	23
26	Nigeria	9	0.51	551	61.22	20
27	Austria	8	0.45	429	53.62	13
28	Egypt	7	0.40	125	17.85	19
29	Portugal	7	0.40	130	18.57	13
30	South Africa	5	0.28	106	21.20	16

Among OECD (Organisation for Economic Co-operation and Development) nations, the United States, the United Kingdom, and Germany stand out as key contributors, as evidenced by their respective total citations of 12,888, 2,419, and 1,961, referencing a combined total of 344, 65, and 61 documents. This trend aligns with the characteristic features of OECD countries, known for their advanced economies, robust research infrastructures, and substantial investments in research and development. The concentration of citations from these nations underscores their pivotal role in shaping and disseminating knowledge on the discussed topics. Furthermore, the prominence of the United States, the United Kingdom, and Germany in citation counts signifies their leadership in driving research and innovation on a global scale, thereby emphasizing the significant contributions of OECD countries to the exchange of scholarly knowledge within the academic community.

### Publication distribution by organization

We did a comprehensive analysis of institutions that have made major contributions to the field of CRISPR-based illness detection. Our research primarily targeted organizations that have published at least three articles in this area. We conducted research involving 5124 organizations, out of which only 23 met the requirement of having written at least five articles. Within this subset, a conspicuous trend became apparent: only two countries exhibited a publishing total of over 10 articles, whereas the rest of the organizations produced fewer than 10 articles each. Out of the 23 organizations that met the required criteria, Chongqing Medical University in China stood out as the top contributor, with 13 published publications and a remarkable citation count of 91, averaging 7 citations per year. The Broad Institute of MIT and Harvard in Cambridge, MA, United States, ranks second with 12 publications and a notable 1418 citations, an average of 118.6 per document from 1992 to 2023. The WYSS Institute for Biologically Inspired Engineering at Harvard University, located in Boston, MA, United States, leads in terms of average citation. It has achieved a remarkable score of 711.60 (TLS = 0) for five articles. The Broad Institute of MIT and Harvard, located in Cambridge, MA, United States, achieved the second rank in this category with an average citation count of 118.16 (TLS = 3) for its twelve documents. Table 2 provides a thorough overview of the top 20 organizations, presenting statistics on the total number of publications, citations, and total link strength.

**TABLE 2:** Organizations that have published at least three studies linked to the identification of diseases using CRISPR technology.

S. No	Organization	Documents	Citations	Average citations	Total link strength
1	Chongqing Medical University, Chongqing, 400016, China	13	91	7	13
2	Chongqing University Three Gorges Hospital, Chongqing, 404000, China	7	17	2.42	12
3	Bioengineering College of Chongqing University, Chongqing, 400044, China	7	10	1.424	12
4	National Facility for Translational Medicine, Shanghai Jiao Tong University, Shanghai, 200240, China	5	27	5.40	9
5	College of Biosystems Engineering and Food Science, Zhejiang University, Hangzhou, 310058, China	6	270	45.00	5
6	College of Laboratory Medicine, Chongqing Medical University, Chongqing, 400016, China	6	25	4.166	5
7	Key Laboratory of On-site Processing Equipment for Agricultural Products, Ministry of Agriculture, Hangzhou, 310058, China	5	256	51.20	5
8	The Center for Clinical Molecular Medical Detection, The First Affiliated Hospital of Chongqing Medical University, Chongqing, 400016, China	6	23	3.83	5
9	Broad Institute of MIT and Harvard, Cambridge, MA, United States	12	1418	118.16	3
10	Howard Hughes Medical Institute, Chevy Chase, MD, United States	6	640	106.66	3
11	Department of Biomedical Engineering, University of Connecticut, Storrs, 06269, CT, United States	6	77	12.83	1
12	Department of Pathology and Laboratory Medicine, University of Connecticut Health Center, Farmington, 06030, CT, United States	6	579	96.50	1
13	Experimental Research Center, Capital Institute of Pediatrics, Beijing	5	25	5.00	0
14	Innovative Genomics Institute, University of California, Berkeley, Berkeley, CA, United States	5	189	106.66	0
15	Jiangsu Institute of Nuclear Medicine, Jiangsu, Wuxi, 214063, China	5	22	4.40	0
16	School of Life Sciences, South China Normal University, Guangzhou, 510631, China	6	567	94.50	0
17	State Key Laboratory of Agricultural Microbiology, Huazhong Agricultural University, Wuhan, 430070, China	5	52	10.40	0
18	University of Chinese Academy of Sciences, Yuquan road, Shijingshan district, Beijing, 100049, China	5	98	19.60	0
19	University of Chinese Academy of Sciences, Beijing, 100049, China	7	61	8.71	0
20	WYSS Institute for Biologically Inspired Engineering, Harvard University, Boston, 02115, MA, United States	5	3558	711.60	0

### Author and co-author relationship

**FIGURE 5:**
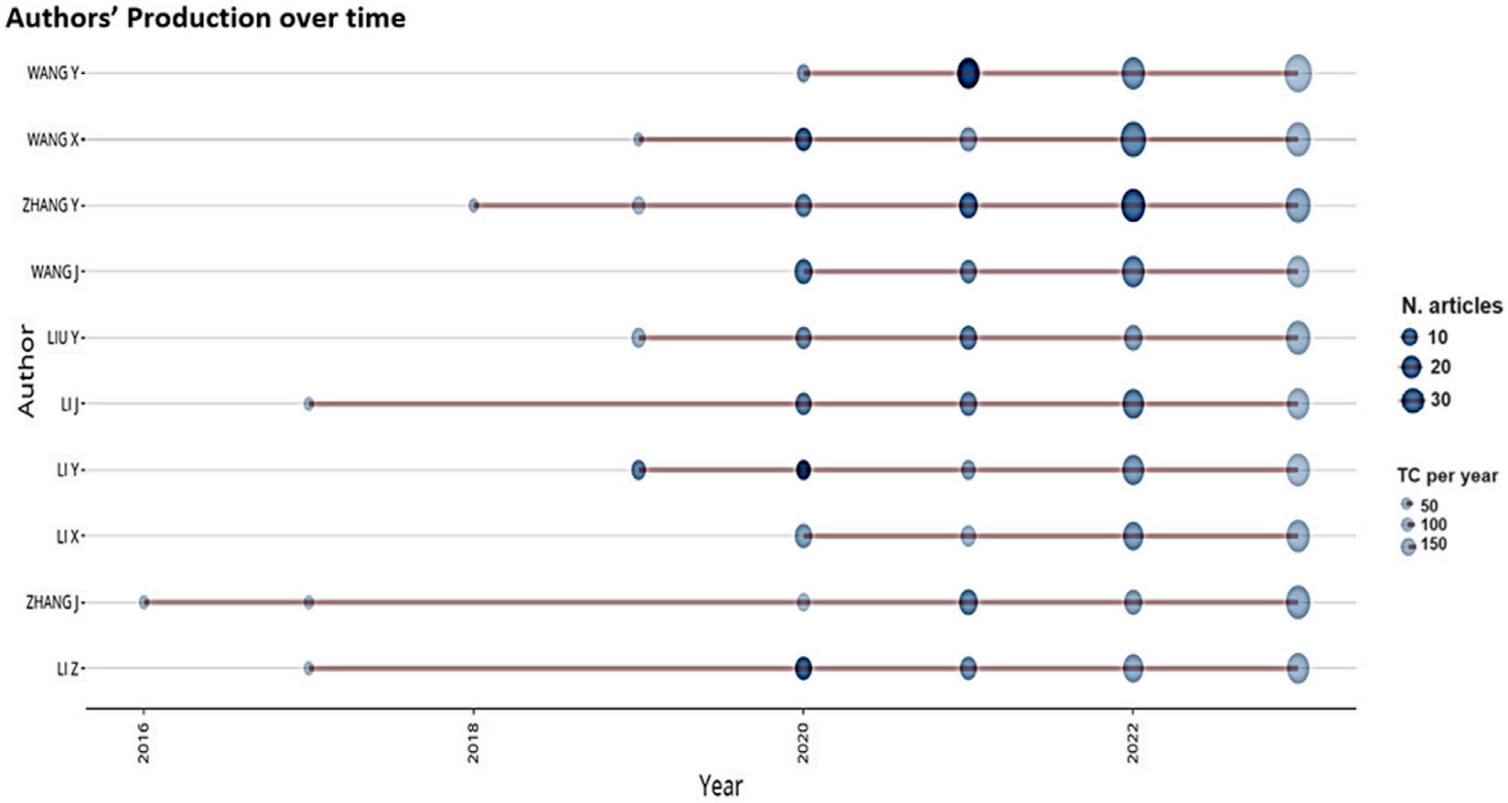
The influence of the top 10 authors in different years is depicted by red lines. The size of the dots represents the amount of publications in different years, while the colour of the dots (varying from light to dark) shows the total number of citations per year (TC).

The assessment of writers who have published articles in the field of CRISPR-based illness detection was conducted by considering the number of published articles and the number of citations received. Among the total of 6357 writers, only 214 individuals have written five or more articles on the topic of CRISPR-based illness detection. According to the information provided in Table 3, Wang H. had the highest number of publications, with a total of 71. Additionally, Wang H. received a total of 1688 citations, and the average number of citations per year was 58.20. Wang X. had the second-highest number of published articles (67) and received 1065 citations, resulting in an average of 15.89 citations per year. Zhang Y., on the other hand, had the third highest number of published articles (63) and received 1248 citations, resulting in an average of 19.80 citations per year. Zhang H. and Li Y. have an average of 35.35 and 34.38 citations each year, respectively. They have a total of 28 and 47 documents, respectively. This places them among the top three in terms of average citations, along with Wang Y. The overall connection strength of the writers, Wang X., Wang Y., and Zhang Y. was analyzed and found to be 117, 106, and 93, respectively. This indicates that these writers excel at collaborating on research about CRISPR-based illness detection with other organizations or countries. From 1992 to 2023, we conducted an examination and analysis of the global evolution of CRISPR-based illness diagnosis (Figure 5). Wang Y achieved the highest publication count (71) during the years 1992 and 2023. Starting in 2020, Wang Y. began intensively researching disease detection using CRISPR technology. In the year 2023, Wang Y. published the maximum number of articles (34) on this topic. Wang X. achieved the second-highest number of published articles (67) between 1992 and 2023 and showed significant activity in 2022 with 27 articles. The author, Zhang J., commenced their work in the field in 2016 and 2017 but subsequently had a significant hiatus from study. They resumed their research activities in 2020 and have been active since then.

**TABLE 3:** The following list comprises the 30 most prominent authors who have published at least five articles on the topic of disease diagnosis using CRISPR technology. The Total link strength in the table represents the overall strength of an author’s co-authorship ties with other researchers.

S. No	Author	Documents	Citations	Average citations	Total link strength
1	Liu, C	29	1688	58.20	42
2	Li, Y	47	1616	34.38	77
3	Zhang, Y	63	1248	19.80	93
4	Wang, Y	71	1120	15.77	106
5	Wang, X	67	1065	15.89	117
6	Li, Z	43	1018	23.67	53
7	Zhang, H	28	990	35.35	52
8	Li, J	48	980	20.41	78
9	Wang, J	50	949	18.98	77
10	Liu, Y	46	824	17.91	68
11	Zhang, J	44	577	13.11	51
12	Zhang, L	24	555	23.12	35
13	Chen, J	24	536	23.33	46
14	Li, X	42	515	12.26	83
15	Liu, J	25	492	19.68	42
16	Huang, X	23	489	21.26	48
17	Chen, Y	34	468	13.76	48
18	Zhang, X	37	467	12.62	71
19	Yang, Y	30	449	14.96	37
20	Zhang, W	29	448	15.44	44
21	Li, C	19	448	23.57	38
22	Wang, H	32	402	12.56	54
23	Zhnag, T	17	396	23.29	26
24	Chen, Z	20	343	17.15	44
25	Chen, X	20	326	16.30	39
26	Liu, X	25	303	12.12	48
27	Wang, C	16	278	17.37	33
28	Li, H	30	212	7.06	43
29	Li, S	17	196	11.52	38
30	Ma, X	17	159	9.35	37

The assessment and analysis of the cooperative endeavors among the authors were carried out by employing TLS measures, as specified in Table 3 and Supplementary Figure S2. After careful investigation, it was found that Wang X. had the highest TLS score of 117, then Wang X., Zhang Y., Li X., and Li J., who had TLS values of 106, 93, 83, and 78, respectively. Given their exceptional networking and collaborative abilities, this discovery suggests that the authors are highly productive as researchers in the field of CRISPR-based illness detection.

### Publication resources

**FIGURE 6:**
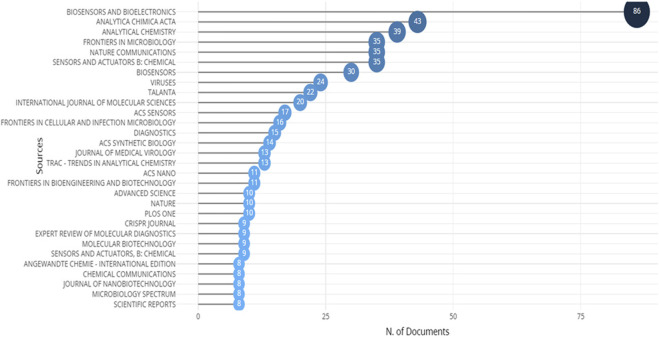
Top journals having research articles published on the topic of disease detection using CRISPR technology, covering the period from 1992 to 2023.

The origin of the article offers valuable understanding regarding the publications that renowned experts choose to disseminate their scientific findings on CRISPR-based illness detection. Table 4 and Figure 6 provide a comprehensive summary of reputable journals that have published research on illness diagnosis using CRISPR technology. According to the analyzed data, Biosensors and Bioelectronics is a highly preferred journal for publication, with a total of 91 articles and 3825 citations. Nature Journal ranked first in terms of average citations, with an average of 183.1 citations per year for only 10 documents. Angewandte Chemie, International Edition Journal, followed with 8 total documents and an average of 97.5 citations per year. The ACS Nano Journal had 11 total documents and an average of 60.36 citations per year. Nature Communications held the second, third, and fourth positions with 35 total documents and an average of 56.94 citations per year. This indicates that there is a lack of notable presence by any one publisher or journal in publishing publications about CRISPR-based illness detection.

**TABLE 4:** Journals that have published three or more articles on the topic of CRISPR-based disease detection between the years 1992 and 2023.

S. No	Journal	Publisher	Documents	Citations	Average citation	Total link strength
1	Biosensors And Bioelectronics	Elsevier	91	3825	42.033	172
2	Analytica Chimica Acta	Elsevier	47	322	6.8511	76
3	Sensors And Actuators B: Chemical	Elsevier	9	363	7.825	12
4	Analytical Chemistry	American Chemical Society (ACS)	39	1321	33.8718	191
5	Frontiers In Microbiology	Frontiers Media SA	35	275	7.8571	29
6	Nature Communications	Nature Research	35	1993	56.9429	17
7	Biosensors	MDPI	30	327	10.9	58
8	Talanta	Elsevier	28	379	13.5357	48
9	Viruses	MDPI	24	836	34.833	8
10	International Journal Of Molecular Sciences	MDPI	20	435	21.75	23
11	Acs Sensors	American Chemical Society (ACS)	17	615	36.176	85
12	Frontiers In Cellular And Infection Microbiology	Frontiers Media SA	16	201	12.562	16
13	Diagnostics	MDPI	15	217	14.466	20
14	Trac - Trends In Analytical Chemistry	Elsevier	15	195	13	38
15	Acs Synthetic Biology	American Chemical Society (ACS)	14	533	38.071	89
16	Journal Of Medical Virology	Wliey	13	89	6.846	11
17	Acs Nano	American Chemical Society (ACS)	11	664	60.363	67
18	Frontiers In Bioengineering And Biotechnology	Frontiers Media SA	11	73	6.636	37
19	Advanced Science	Wiley	10	231	23.1	0
20	Nature	Nature Research	10	1831	183.1	3
21	Plos One	Public Library of Science (PLOS)	10	192	19.2	1
22	Crispr Journal	Mary Ann Liebert, Inc	9	60	6.666	27
23	Expert Review Of Molecular Diagnostics	Taylor and Francis	9	90	10	14
24	Molecular Biotechnology	Springer	9	50	5.555	13
25	Sensors And Actuators, B: Chemical	Elsevier	9	363	40.333	12
26	Angewandte Chemie - International Edition	Wiley	8	780	97.5	124
27	Chemical Communications	Royal Society of Chemistry (RSC)	8	92	11.5	19
28	Journal Of Nanobiotechnology	Springer	8	130	16.25	7
29	Microbiology Spectrum	American Society of Microbiology (ASM)	8	29	3.625	9
30	Scientific Reports	Nature Research	8	101	12.625	4

The analysis also revealed a statistically significant positive correlation, indicating that journals with APCs (Article Processing Charges) tend to have higher citation counts for the articles they publish. However, it's important to recognize that correlation does not imply causation. The relationship between citation count and APCs may be influenced by other factors, such as the open-access policies associated with APCs. Indeed, APCs often lead to open-access publication, making articles more readily accessible to a wider audience. This increased accessibility can enhance the visibility of the paper, leading to greater citations over time. It's crucial to consider the role of open access policies in driving article visibility and subsequent citation counts.

For bibliometric analysis, we also examine whether there is a correlation between citation count and the impact factor of journals. The top five journals with citations and impact factor are Biosensors and Bioelectronics with 3825 citations and an impact factor of 12.6, Nature Communications with 1993 citations and an impact factor of 16.6, Nature with 1831 citations and an impact factor of 64.8, Analytical Chemistry with 1321 citations and an impact factor of 7.4, and Viruses with 836 citations and an impact factor of 4.7. Generally, there is often a positive correlation between citation count and impact factor of journals. Journals with higher impact factors tend to attract more citations because they are perceived as publishing higher-quality research and are more widely read within the academic community. However, it's important to note that correlation does not necessarily imply causation, and other factors such as field of study, publication frequency, and editorial policies can also influence citation counts.

### Document and citation relationship

**FIGURE 7:**
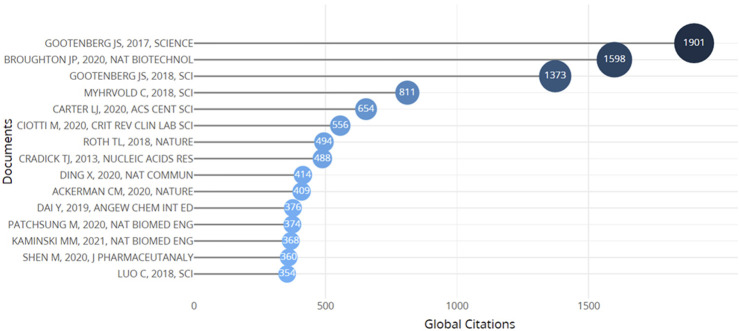
Below are the graph of top 15 article’s authors in the field of CRISPR-based disease detection that have received at least 100 citations.

The evaluation of a publication’s quantity and its importance among readers is ascertained through journal articles and the number of citations they acquire. In general, a significant number of citations reflects the extent to which works are acknowledged by other authors or experts in the same subject (Bornmann and Marx, 2015). To conduct the study, a minimum threshold of ≥100 citations was set. Out of the 1436 articles that were published, only 80 articles satisfied this requirement. The data indicates that the work written by Broughton, titled “CRISPR–Cas12-based detection of SARS-CoV-2,” and published in 2020, had the most citations (1698) until 2023 (Table 5; Figure 7). Broughton’s research focused on the origin of a beta coronavirus called SARS-CoV-2, which appeared in Wuhan, China, in December 2019 and subsequently caused the worldwide COVID-19 pandemic. The study described the creation of a fast (<40 min), easily applicable, and precise CRISPR–Cas12-based lateral flow test for identifying SARS-CoV-2 in respiratory swab RNA extracts. The validation process involved the use of artificially created reference samples and actual clinical samples from the United States. These samples were taken from 36 COVID-19 patients and 42 individuals who had other viral respiratory infections. The results demonstrated a fast and visually based alternative to the CDC’s SARS-CoV-2 real-time RT-PCR assay. The findings exhibited a 95% positive predictive concordance and a 100% negative predictive concordance (Broughton et al., 2020). The total citations for a research article indicate the frequency with which the piece has been cited or utilized in the work of others within the same subject. Consequently, as the citation number of an article increases, so does its impact on the research area, making it more influential. Additionally, this phenomenon demonstrates the increasing prominence of the research field, as a growing number of authors and researchers are actively involved in conducting studies and publishing their findings, resulting in heightened recognition and citations. Additionally, the average annual citation count that articles acquire also influences their popularity. The most-cited articles each year are by Broughton et al. (2020), which are called “CRISPR-Cas12-based detection of SARS-CoV-2,” Gootenberg et al. (2017), which is called “Nucleic acid detection with CRISPR-Cas13a/C2c2,” and Gootenberg et al. (2018), which is called “Multiplexed and portable nucleic acid detection platform with Cas13, Cas12a, and Csm6.” and Gootenberg et al. (2018) titled “Multiplexed and portable nucleic acid detection platform with Cas13, Cas12a, and Csm6” are the most highly cited articles on average per year. This indicates that the articles had a substantial and enduring influence on the scientific community.

**TABLE 5:** Here are the top 15 articles in the field of CRISPR-based disease detection that have received at least 100 citations.

S. No	Document	Citations	Average citation per year	Links	References
1	Nucleic acid detection with CRISPR-Cas13a/C2c2	1901	237.6	0	Gootenberg et al. (2017)
2	CRISPR–Cas12-based detection of SARS-CoV-2	1598	319.6	1	Broughton et al. (2020)
3	Multiplexed and portable nucleic acid detection platform with Cas13, Cas12a and Csm6	1373	196.1	0	Gootenberg et al. (2018)
4	Field-deployable viral diagnostics using CRISPR-Cas13	811	115.8	20	Myhrvold et al. (2018)
5	Assay Techniques and Test Development for COVID-19 Diagnosis	654	130.8	0	Carter et al. (2020)
6	The COVID-19 pandemic	556	111.2	0	Ciotti et al. (2020)
7	Reprogramming human T cell function and specificity with non-viral genome targeting	494	70.57	0	Roth et al. (2018)
8	CRISPR/Cas9 systems targeting β-globin and CCR5 genes have substantial off-target activity	488	40.66	0	Cradick et al. (2013)
9	Ultrasensitive and visual detection of SARS-CoV-2 using all-in-one dual CRISPR-Cas12a assay	414	82.8	0	Ding et al. (2020)
10	Massively multiplexed nucleic acid detection with Cas13	409	81.8	1	Ackerman et al. (2020)
11	Exploring the Trans-Cleavage Activity of CRISPR-Cas12a (cpf1) for the Development of a Universal Electrochemical Biosensor	376	62.6	6	Dai et al. (2019)
12	Clinical validation of a Cas13-based assay for the detection of SARS-CoV-2 RNA	374	74.8	4	Patchsung et al. (2020)
13	CRISPR-based diagnostics	368	92	2	Kaminski et al. (2021)
14	Recent advances and perspectives of nucleic acid detection for coronavirus	360	72	1	Shen et al. (2020)
15	Dynamic DNA methylation: In the right place at the right time	354	50.5	0	Luo et al. (2018)

## Concurrence with author keywords

Analysing keyword co-occurrence greatly aids in identifying topics and following the development of trends in research (Lozano et al., 2019). This analysis is crucial in communicating the main themes of scientific works and defining specific areas of study. Keywords serve as a navigational tool for readers who are actively examining information pertaining to a specific subject. This facilitates the identification and incorporation of relevant works within the domain of literary studies. An essential aspect of keywords is their capacity to augment the overall visibility of research articles, especially through extensively employed internet search engines like Scopus, Web of Science, PubMed, and Google Scholar databases. This not only simplifies the search procedure for other scholars but also adds to the growth of the current knowledge base. Therefore, researchers must carefully choose the most suitable keywords to ensure that their work receives widespread readership, references, and recognition in their respective domains. The examination primarily examined keywords that appeared 25 or more times out of a total of 3170 occasions. Only 21 terms satisfied this frequency criteria. The most used keywords in the field of CRISPR-based illness detection are SARS-CoV-2, COVID-19, CRISPR, diagnosis, diagnostic, nucleic acid detection, biosensors, CRISPR-Cas, CRISPR/Cas12a, and detection. The frequencies of these keywords were 211, 177, 163, 48, 51, 59, 47, 49, 126, and 34, as indicated in Table 6 and Supplementary Figure S3. The results of our study reveal a significant focus on SARS-CoV-2, COVID-19, and CRISPR in relation to the use of CRISPR for disease detection. Significantly, the time span between 2019 and 2021 witnessed significant scientific advancements in tackling the COVID-19 pandemic. Extensive research activities were undertaken from late 2019 to 2021 in reaction to the advent of the new coronavirus SARS-CoV-2 and the ensuing worldwide health crisis. The initial efforts were centred on characterising the virus and comprehending its transmission dynamics. In 2020 and 2021, there was a focused and swift push to develop and distribute numerous COVID-19 vaccines worldwide. In addition to immunisation techniques, researchers investigated therapeutic interventions and antiviral drugs to reduce the severity of the disease and alleviate symptoms. At the same time, it became clear that CRISPR technology was very important for speeding up the development of diagnostic tools for finding SARS-CoV-2 through quick and accurate testing methods. Scientists tested how well CRISPR could edit genes for possible medical uses. They focused on editing the viral genome to stop the spread of SARS-CoV-2. Furthermore, CRISPR technology was utilized to clarify the influence of genetic elements on the severity of diseases, as demonstrated by research investigating the contribution of host genetics to the susceptibility to COVID-19. The primary aim of this collaborative and interdisciplinary research method was to thoroughly tackle the issues presented by the epidemic. This means that pointing out unclear areas helps us see where information might be lacking, which leads to a better understanding of how widely CRISPR-based illness diagnosis is used.

**TABLE 6:** Keywords in the field of CRISPR-based disease detection.

S. No	Keyword	Occurrences	Total link strength
1	SARS-CoV-2	211	316
2	COVID-19	177	289
3	CRISPR	163	187
4	Diagnosis	48	85
5	Diagnostic	51	85
6	Nucleic acid detection	59	78
7	Biosensors	47	72
8	CRISPR-Cas	49	59
9	CRISPR/Cas12a	126	52
10	Detection	38	52
11	Isothermal amplification	34	45
12	Cas12a	31	44
13	CRISPR-Cas12a	68	42
14	CRISPR/Cas	47	41
15	Molecular diagnostics	25	39
16	Biosensor	35	38
17	RPA	25	34
18	Recombinase polymerase amplification	27	29
19	Genome editing	28	28
20	CRISPR/cas9	49	15
21	CRISPR/Cas13a	28	14

### Three-field plot analysis

**FIGURE 8:**
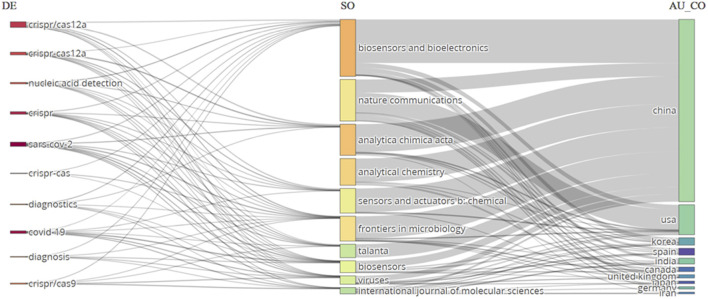
A graph depicting the correlation between Author keywords (DE), Sources (SO), and Countries (AU_CO) was produced by a three-field plot analysis. The graph displays lines and boxes that depict the association among the three variables. The thickness of the lines and the height of the boxes indicate the level of correlation between the variables.

Bibliometric studies utilise a three-field plot analysis to comprehensively evaluate academic entities such as authors, journals, or institutions (Kumar and Goel, 2022). This technique employs a graphical representation to incorporate three crucial indicators: productivity (output), impact (citation), and collaboration (co-authorship). Entities with more citations are placed higher up on the vertical axis. Higher numbers towards the right suggest greater output on the horizontal axis, representing productivity. Additionally, larger markers indicate higher levels of collaboration, representing the magnitude of the markers and the level of collaboration. This visual method facilitates the assessment of the overall performance of entities within a specific study subject by utilising comparisons and benchmarking. Entities located in the upper-right quadrant are often characterised by high production and impact, indicating a substantial level of influence. On the other hand, the lower-left quadrant is associated with lower productivity and impact. We conducted an analysis on the link between keywords, sources, and nations using a three-field plot created with Biblioshiny in RStudio (Figure 8). The plot reveals a strong correlation between CRISPR/Cas12a and Biosensors and Bioelectronics, Analytica Chimica Acta, and Sensors and Actuators B: Chemical Journals in China, the USA, Korea, India, and Canada. The analysis would also recognize the scientific areas where there is a lack of representation from certain countries, which presents an opportunity for additional research and collaboration.

**FIGURE 9:**
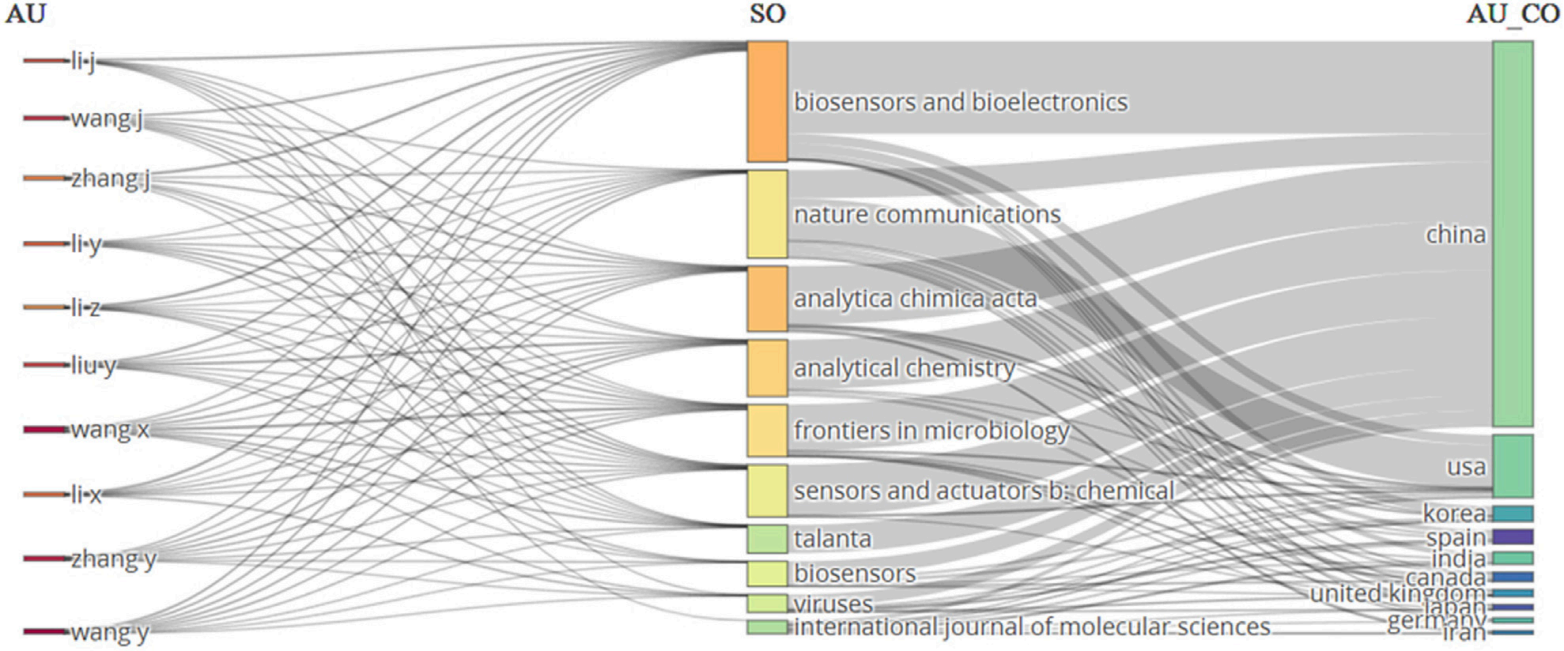
A graph depicting the correlation between Authors (AU), Sources (SO), and Countries (AU_CO) was produced by a three-field plot analysis. The graph displays lines and boxes that depict the association among the three variables. The thickness of the lines and the height of the boxes indicate the level of correlation between the variables.

Another three-field plot between the authors, journals, and author countries was generated using Biblioshiny in RStudio (Figure 9). The plot reveals a strong correlation between author Li Y., the journal Biosensors and Bioelectronics, and country China for CRISPR-based studies for disease detection. The journal Biosensors and Bioelectronics, characterized by 10 incoming and 8 outgoing flows, reflects a robust exchange of citations, indicating its significant influence within the scholarly community. Incoming flows signify citations from external sources to articles published in the journal, showcasing its widespread recognition and contribution to the field, while outgoing flows represent citations made by the journal’s articles to external sources, demonstrating its engagement with diverse literature. Author Li J’s 7 outgoing flow counts suggest a thorough engagement with existing scholarship, indicating a broad knowledge base and comprehensive understanding of the subject matter. China’s prominence, with 10 incoming flow counts, underscores its active participation in global research, with Chinese researchers frequently cited across various journals, authors, and countries, indicating a substantial contribution to scholarly discourse on an international scale. These findings highlight the interconnectedness and significance of scholarly engagement and citation practices within the academic community.

## Limitations

Analysing bibliometrics is a powerful method for examining trends and advancements in the field of science. However, like any analytical approach, bibliometric analysis is not without certain limitations that require careful attention when interpreting the results. The effectiveness of bibliometric analysis depends on the quality of the available metadata. Insufficient or inconsistent information can make it difficult to accurately determine the origins, keywords, and countries associated with a particular study. Bibliometric analysis is also prone to biases in the selection of sources and databases. For instance, particular journals may have a higher inclination towards publishing research about CRISPR-based disease detection compared to others, or specific nations may be disproportionately represented in the databases utilized for the analysis. This can result in an inadequate or misrepresented depiction of the subject.

## Conclusion

The present study focused on analysis of trends in research publications withing the field of CRISPR-based disease detection using bibliometric analysis. We acquired a dataset of 1407 after eliminating duplicates using Zotero reference manager obtained from the Scopus database. The analysis was carried out to explore trends in publication pattern and networks. It revealed that a significant rise was seen post-2015 with China, Unites States, India, United Kingdom, and Germany being top five countries with highest publication status in the field. Among these countries, organizations like Chongqing Medical University of China, The Broad Institute of MIT and Harvard of United States, and the WYSS Institute for Biologically Inspired Engineering at Harvard of Boston, United States were listed as top three organizations actively involved in the field of CRISPR-based disease detection. The publication by Broughton in 2020, Gootenberg in 2017, and 2018 received the highest citation number and total link strength (TLS), and Journal Biosensors and Bioelectronics holds the highest publication number in this field. Our analysis also revealed that breast cancer, HIV, MRSA (Methicillin-resistant *Staphylococcus aureus*), prostate cancer, and acute leukemia are among the most prevalent diseases associated with CRISPR research. Understanding these associations can indeed inform policymakers in the healthcare domain about potential courses of action. For instance, it highlights the urgent need for continued research and development of CRISPR-based therapies targeting these diseases. Additionally, it underscores the importance of allocating resources and funding towards supporting research initiatives aimed at addressing these prevalent health concerns. By prioritizing these areas, policymakers can facilitate the advancement of CRISPR technologies in tackling some of the most pressing medical challenges of our time.

All these analyses provide valuable and significant insight for the researchers, policymakers, funding agencies, and institutions about the state of the field, and collaborative opportunities for the future. Another promising development for the prospect is the use of many other machine learning and artificial intelligence to analyze bibliometric datasets. Another promising tool for bibliometric analysis is the social media. Social media data is expected to have a significant impact on future bibliometric trends. Altmetrics, derived from platforms such as Twitter and LinkedIn, encompass social media mentions and engagement metrics. They offer a dynamic evaluation of a publication’s influence that goes beyond conventional citation measures. Conducting social network analysis on these platforms allows us to identify patterns of collaboration while monitoring hashtag movements provides valuable information about upcoming study subjects. Conducting sentiment analysis on social media enhances comprehension of public sentiment. As researchers continue to utilize social media for sharing and working together, evaluating the effectiveness of communication, and discovering possible collaborators through online interactions have become essential elements of comprehensive bibliometric analysis. The integration of bibliometrics and social media data is anticipated to shape future trends by offering a holistic perspective on research effects, dissemination techniques, and collaborative possibilities.

## Data Availability

The original contributions presented in the study are included in the article/Supplementary Material, further inquiries can be directed to the corresponding authors.
