# Supplementary material for: Bibliometric analysis on CRISPR/Cas: a potential Sherlock Holmes for disease detection
Source: Front Mol Biosci. 2024 Jul 11;11:1383268. doi: 10.3389/fmolb.2024.1383268 (PMC11269658; doi:10.3389/fmolb.2024.1383268)
Supplement: Supplementary file 1 [file Table1.docx]

| **Supplementary Table 1:** Countries with frequency of publications in CRISPR-based disease detection |
| --- |
| \| **Countries** \| **Frequency of CRISPR-based disease detection publications** \| \| --- \| --- \| \| CHINA \| 5200 \| \| USA \| 2253 \| \| INDIA \| 466 \| \| GERMANY \| 302 \| \| SOUTH KOREA \| 263 \| \| UK \| 247 \| \| IRAN \| 204 \| \| CANADA \| 176 \| \| JAPAN \| 174 \| \| SPAIN \| 160 \| \| THAILAND \| 151 \| \| FRANCE \| 116 \| \| ITALY \| 109 \| \| SAUDI ARABIA \| 102 \| \| BRAZIL \| 97 \| \| NETHERLANDS \| 91 \| \| AUSTRALIA \| 89 \| \| SWITZERLAND \| 60 \| \| MALAYSIA \| 56 \| \| PAKISTAN \| 55 \| \| SINGAPORE \| 55 \| \| TURKEY \| 53 \| \| POLAND \| 47 \| \| SWEDEN \| 43 \| \| BANGLADESH \| 37 \| \| PERU \| 33 \| \| MEXICO \| 32 \| \| BELGIUM \| 31 \| \| KENYA \| 27 \| \| AUSTRIA \| 25 \| \| IRELAND \| 21 \| \| CZECH REPUBLIC \| 20 \| \| NIGERIA \| 20 \| \| MOROCCO \| 19 \| \| QATAR \| 19 \| \| ARGENTINA \| 18 \| \| TANZANIA \| 17 \| \| DENMARK \| 16 \| \| PORTUGAL \| 16 \| \| INDONESIA \| 15 \| \| ROMANIA \| 14 \| \| EGYPT \| 12 \| \| LITHUANIA \| 12 \| \| UNITED ARAB EMIRATES \| 12 \| \| KAZAKHSTAN \| 11 \| \| SOUTH AFRICA \| 11 \| \| CHILE \| 9 \| \| NEW ZEALAND \| 9 \| \| SLOVAKIA \| 9 \| \| GREECE \| 8 \| \| ECUADOR \| 7 \| \| NEPAL \| 7 \| \| AZERBAIJAN \| 6 \| \| HUNGARY \| 6 \| \| NORWAY \| 6 \| \| RWANDA \| 5 \| \| UGANDA \| 5 \| \| GUINEA \| 4 \| \| IRAQ \| 4 \| \| SWAZILAND \| 4 \| \| CENTRAL AFRICAN REPUBLIC \| 3 \| \| HONDURAS \| 3 \| \| ISRAEL \| 3 \| \| OMAN \| 3 \| \| PAPUA NEW GUINEA \| 3 \| \| SIERRA LEONE \| 3 \| \| SLOVENIA \| 3 \| \| UKRAINE \| 3 \| \| DOMINICAN REPUBLIC \| 2 \| \| ESTONIA \| 2 \| \| ICELAND \| 2 \| \| SUDAN \| 2 \| \| CAMEROON \| 1 \| \| ETHIOPIA \| 1 \| \| FINLAND \| 1 \| \| LEBANON \| 1 \| \| PHILIPPINES \| 1 \| \| SRI LANKA \| 1 \| \| VENEZUELA \| 1 \| \| YEMEN \| 1 \| |
