# Supplementary material for: Bibliometric analysis on CRISPR/Cas: a potential Sherlock Holmes for disease detection
Source: Front Mol Biosci. 2024 Jul 11;11:1383268. doi: 10.3389/fmolb.2024.1383268 (PMC11269658; doi:10.3389/fmolb.2024.1383268)
Supplement: Supplementary file 2 [file Image2.pdf]

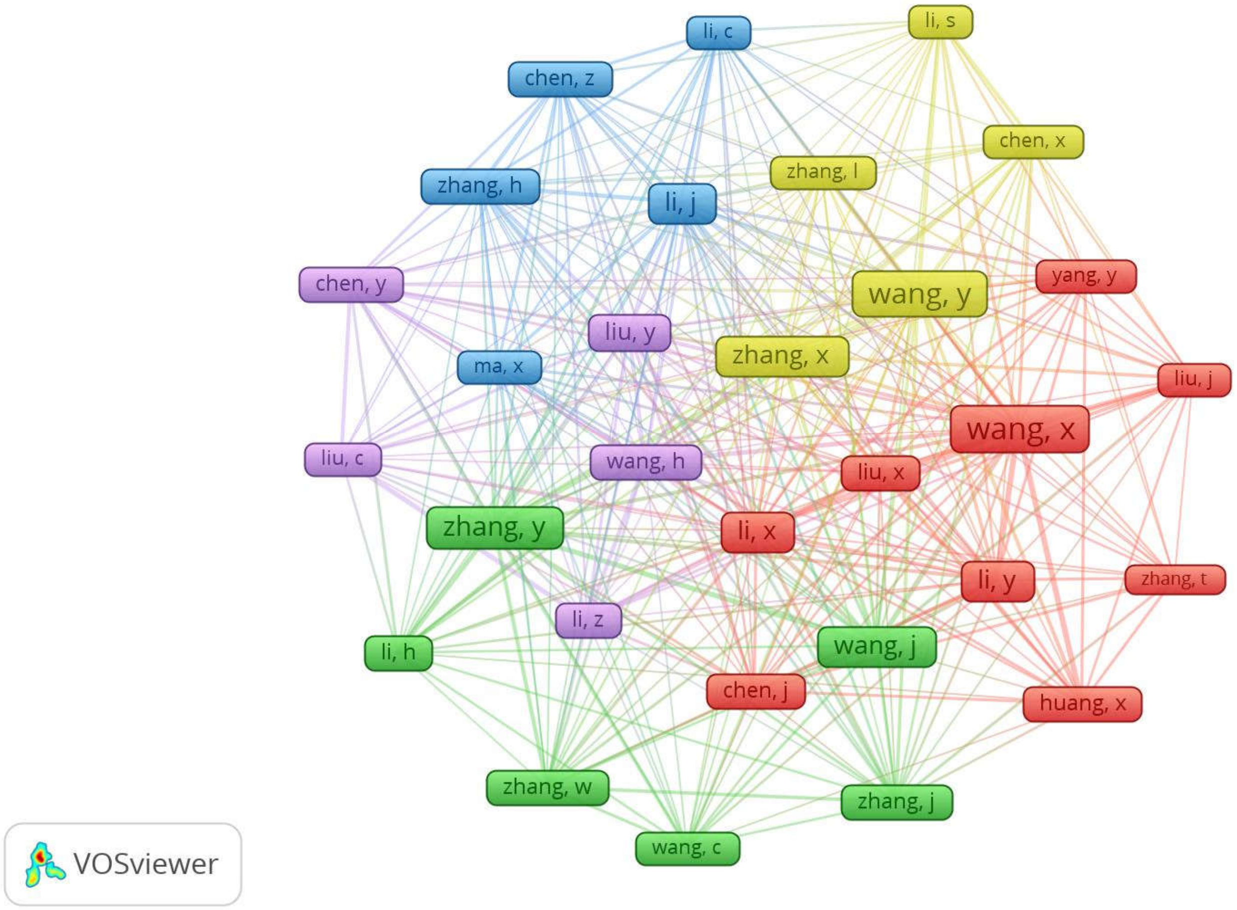

Fig S2: A network visualization was created to represent the collaborative research conducted by writers on CRISPR-based illness detection. Only authors with at least five publications were included in the analysis. The various hues in the diagram symbolize distinct groupings. A cluster refers to a group of interconnected nodes or items that are tightly associated with each other. Every object within a network is allocated to a single cluster. The color of an object is dictated by the cluster it is associated with. The lines connecting objects symbolize hyperlinks, while the proximity between two items roughly signifies their level of correlation.
