# Supplementary figures and images for: Bibliometric analysis on CRISPR/Cas: a potential Sherlock Holmes for disease detection
Source: Front Mol Biosci. 2024 Jul 11;11:1383268. doi: 10.3389/fmolb.2024.1383268 (PMC11269658; doi:10.3389/fmolb.2024.1383268)

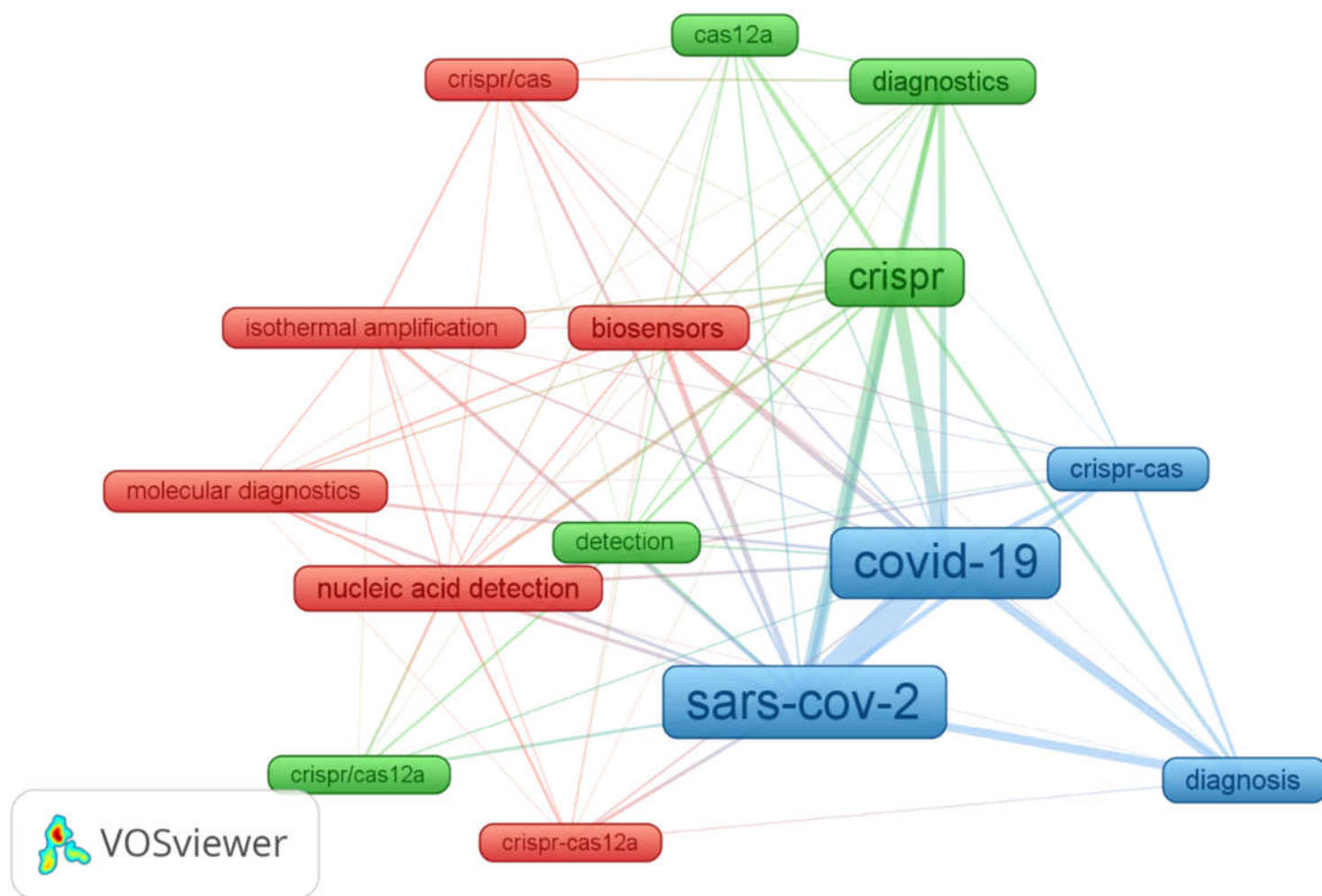

**Fig S3:** A network visualization was created using keywords that appeared at least 25 times.

Supplement: Supplementary file 3 [file Image3.pdf]
