# Supplementary material for: Bibliometric analysis on CRISPR/Cas: a potential Sherlock Holmes for disease detection
Source: Front Mol Biosci. 2024 Jul 11;11:1383268. doi: 10.3389/fmolb.2024.1383268 (PMC11269658; doi:10.3389/fmolb.2024.1383268)
Supplement: Supplementary file 4 [file Table2.docx]

| **Supplementary Table 2:** Countries collaboration along with frequency of publications in CRISPR-based disease detection |
| --- |
| \| **From** \| **To** \| **Frequency of publication** \| \| --- \| --- \| --- \| \| AUSTRALIA \| BANGLADESH \| 1 \| \| DENMARK \| 1 \| \| GUINEA \| 1 \| \| HONG KONG \| 1 \| \| MALAYSIA \| 4 \| \| NEW ZEALAND \| 1 \| \| PAPUA NEW GUINEA \| 1 \| \| PHILIPPINES \| 1 \| \| POLAND \| 1 \| \| SINGAPORE \| 1 \| \| SOUTH AFRICA \| 1 \| \| SWITZERLAND \| 1 \| \| TANZANIA \| 1 \| \| TURKEY \| 1 \| \| UGANDA \| 1 \| \| ISRAEL \| 1 \| \| NORWAY \| 1 \| \| AZERBAIJAN \| UKRAINE \| 1 \| \| BANGLADESH \| EGYPT \| 1 \| \| LEBANON \| 1 \| \| MEXICO \| 1 \| \| NIGERIA \| 2 \| \| UGANDA \| 1 \| \| UKRAINE \| 1 \| \| BELGIUM \| AUSTRIA \| 2 \| \| CZECH REPUBLIC \| 1 \| \| GREECE \| 1 \| \| KENYA \| 1 \| \| NORWAY \| 1 \| \| BRAZIL \| AUSTRALIA \| 1 \| \| CHILE \| 1 \| \| HONDURAS \| 1 \| \| PERU \| 1 \| \| PHILIPPINES \| 1 \| \| UGANDA \| 1 \| \| CANADA \| AUSTRALIA \| 3 \| \| BRAZIL \| 1 \| \| DENMARK \| 1 \| \| ITALY \| 4 \| \| JAPAN \| 1 \| \| MALAYSIA \| 2 \| \| PORTUGAL \| 1 \| \| SINGAPORE \| 1 \| \| SOUTH AFRICA \| 1 \| \| SPAIN \| 1 \| \| SWEDEN \| 1 \| \| THAILAND \| 1 \| \| TURKEY \| 1 \| \| CHINA \| AUSTRALIA \| 10 \| \| BANGLADESH \| 1 \| \| CANADA \| 10 \| \| CENTRAL AFRICAN REPUBLIC \| 1 \| \| DENMARK \| 2 \| \| EGYPT \| 1 \| \| FINLAND \| 1 \| \| FRANCE \| 1 \| \| GERMANY \| 3 \| \| GUINEA \| 1 \| \| HONG KONG \| 9 \| \| INDIA \| 3 \| \| INDONESIA \| 1 \| \| IRAN \| 1 \| \| ITALY \| 4 \| \| JAPAN \| 4 \| \| KENYA \| 5 \| \| KOREA \| 8 \| \| MALAYSIA \| 2 \| \| NETHERLANDS \| 1 \| \| PAKISTAN \| 4 \| \| PAPUA NEW GUINEA \| 1 \| \| PERU \| 1 \| \| POLAND \| 1 \| \| PORTUGAL \| 1 \| \| RWANDA \| 4 \| \| SAUDI ARABIA \| 9 \| \| SINGAPORE \| 3 \| \| SPAIN \| 1 \| \| SWAZILAND \| 1 \| \| SWEDEN \| 2 \| \| SWITZERLAND \| 1 \| \| THAILAND \| 3 \| \| UNITED ARAB EMIRATES \| 1 \| \| UNITED KINGDOM \| 14 \| \| USA \| 61 \| \| CZECH REPUBLIC \| GREECE \| 1 \| \| ISRAEL \| 1 \| \| DENMARK \| ESTONIA \| 1 \| \| SOUTH AFRICA \| 1 \| \| EGYPT \| HUNGARY \| 1 \| \| LEBANON \| 1 \| \| SAINT KITTS AND NEVIS \| 1 \| \| UGANDA \| 1 \| \| UKRAINE \| 1 \| \| FRANCE \| ARGENTINA \| 1 \| \| BRAZIL \| 1 \| \| CENTRAL AFRICAN REPUBLIC \| 1 \| \| CZECH REPUBLIC \| 1 \| \| GUINEA \| 1 \| \| ISRAEL \| 1 \| \| ITALY \| 1 \| \| SAUDI ARABIA \| 1 \| \| SWITZERLAND \| 1 \| \| GERMANY \| AUSTRIA \| 7 \| \| BELGIUM \| 1 \| \| CANADA \| 1 \| \| DENMARK \| 2 \| \| EGYPT \| 1 \| \| ESTONIA \| 1 \| \| FRANCE \| 2 \| \| IRAN \| 1 \| \| IRELAND \| 1 \| \| ISRAEL \| 1 \| \| ITALY \| 3 \| \| KENYA \| 1 \| \| MEXICO \| 1 \| \| NETHERLANDS \| 5 \| \| PAKISTAN \| 1 \| \| POLAND \| 1 \| \| QATAR \| 1 \| \| SPAIN \| 1 \| \| SWEDEN \| 1 \| \| SWITZERLAND \| 3 \| \| TURKEY \| 2 \| \| UNITED KINGDOM \| 6 \| \| GUINEA \| PAPUA NEW GUINEA \| 1 \| \| HUNGARY \| SAINT KITTS AND NEVIS \| 1 \| \| INDIA \| AUSTRALIA \| 2 \| \| AUSTRIA \| 1 \| \| BANGLADESH \| 2 \| \| CANADA \| 1 \| \| CZECH REPUBLIC \| 1 \| \| DENMARK \| 1 \| \| EGYPT \| 3 \| \| FRANCE \| 1 \| \| GERMANY \| 1 \| \| HUNGARY \| 1 \| \| INDONESIA \| 1 \| \| IRAN \| 1 \| \| IRELAND \| 1 \| \| ISRAEL \| 2 \| \| ITALY \| 2 \| \| JAPAN \| 2 \| \| KOREA \| 3 \| \| LEBANON \| 1 \| \| MALAYSIA \| 3 \| \| MOROCCO \| 1 \| \| NETHERLANDS \| 1 \| \| NIGERIA \| 2 \| \| SAINT KITTS AND NEVIS \| 1 \| \| SAUDI ARABIA \| 2 \| \| SINGAPORE \| 1 \| \| SLOVENIA \| 1 \| \| SOUTH AFRICA \| 1 \| \| SPAIN \| 1 \| \| SRI LANKA \| 1 \| \| SWITZERLAND \| 1 \| \| TURKEY \| 1 \| \| UGANDA \| 1 \| \| UKRAINE \| 1 \| \| UNITED KINGDOM \| 1 \| \| IRAN \| AUSTRALIA \| 2 \| \| AZERBAIJAN \| 2 \| \| BELGIUM \| 2 \| \| CANADA \| 4 \| \| CZECH REPUBLIC \| 1 \| \| DENMARK \| 2 \| \| ETHIOPIA \| 1 \| \| ITALY \| 3 \| \| MALAYSIA \| 1 \| \| NETHERLANDS \| 1 \| \| POLAND \| 1 \| \| PORTUGAL \| 2 \| \| QATAR \| 1 \| \| SINGAPORE \| 2 \| \| SOUTH AFRICA \| 1 \| \| SWEDEN \| 1 \| \| TURKEY \| 4 \| \| UKRAINE \| 1 \| \| IRELAND \| EGYPT \| 1 \| \| PORTUGAL \| 1 \| \| SLOVENIA \| 1 \| \| ITALY \| AUSTRALIA \| 3 \| \| BANGLADESH \| 1 \| \| BELGIUM \| 1 \| \| DENMARK \| 2 \| \| EGYPT \| 1 \| \| ESTONIA \| 1 \| \| GREECE \| 1 \| \| HUNGARY \| 1 \| \| LEBANON \| 1 \| \| MALAYSIA \| 3 \| \| NETHERLANDS \| 1 \| \| NIGERIA \| 1 \| \| PORTUGAL \| 1 \| \| SINGAPORE \| 1 \| \| SOUTH AFRICA \| 1 \| \| SWITZERLAND \| 1 \| \| TURKEY \| 1 \| \| UGANDA \| 1 \| \| UKRAINE \| 1 \| \| JAPAN \| EGYPT \| 1 \| \| FRANCE \| 1 \| \| HUNGARY \| 1 \| \| MOROCCO \| 1 \| \| SAINT KITTS AND NEVIS \| 1 \| \| SWITZERLAND \| 1 \| \| KENYA \| AUSTRIA \| 1 \| \| NORWAY \| 1 \| \| RWANDA \| 1 \| \| SWAZILAND \| 1 \| \| KOREA \| BANGLADESH \| 1 \| \| CANADA \| 1 \| \| CZECH REPUBLIC \| 2 \| \| INDONESIA \| 1 \| \| IRAN \| 2 \| \| ITALY \| 1 \| \| JAPAN \| 2 \| \| PORTUGAL \| 1 \| \| SINGAPORE \| 1 \| \| SPAIN \| 1 \| \| SWITZERLAND \| 1 \| \| UNITED KINGDOM \| 1 \| \| MALAYSIA \| BANGLADESH \| 2 \| \| DENMARK \| 1 \| \| IRAQ \| 1 \| \| NIGERIA \| 1 \| \| SINGAPORE \| 2 \| \| SOUTH AFRICA \| 1 \| \| TURKEY \| 2 \| \| MEXICO \| DOMINICAN REPUBLIC \| 1 \| \| ECUADOR \| 1 \| \| MOROCCO \| EGYPT \| 1 \| \| HUNGARY \| 1 \| \| SAINT KITTS AND NEVIS \| 1 \| \| NETHERLANDS \| AUSTRIA \| 1 \| \| BANGLADESH \| 1 \| \| BELGIUM \| 1 \| \| EGYPT \| 1 \| \| LEBANON \| 1 \| \| NIGERIA \| 1 \| \| POLAND \| 1 \| \| QATAR \| 1 \| \| SWEDEN \| 1 \| \| SWITZERLAND \| 1 \| \| UGANDA \| 1 \| \| UKRAINE \| 1 \| \| NIGERIA \| EGYPT \| 1 \| \| LEBANON \| 1 \| \| SIERRA LEONE \| 1 \| \| SOUTH AFRICA \| 1 \| \| UGANDA \| 1 \| \| UKRAINE \| 1 \| \| NORWAY \| ICELAND \| 1 \| \| PAKISTAN \| QATAR \| 1 \| \| UNITED ARAB EMIRATES \| 1 \| \| PERU \| ARGENTINA \| 1 \| \| PORTUGAL \| AZERBAIJAN \| 1 \| \| UKRAINE \| 1 \| \| SAUDI ARABIA \| BANGLADESH \| 1 \| \| OMAN \| 1 \| \| PAKISTAN \| 1 \| \| SINGAPORE \| 1 \| \| UNITED ARAB EMIRATES \| 1 \| \| SINGAPORE \| CZECH REPUBLIC \| 1 \| \| DENMARK \| 1 \| \| SOUTH AFRICA \| 1 \| \| TURKEY \| 1 \| \| SPAIN \| BANGLADESH \| 1 \| \| BELGIUM \| 1 \| \| BRAZIL \| 1 \| \| EGYPT \| 1 \| \| FRANCE \| 2 \| \| ITALY \| 2 \| \| LEBANON \| 1 \| \| NETHERLANDS \| 1 \| \| NIGERIA \| 1 \| \| SWEDEN \| 1 \| \| SWITZERLAND \| 1 \| \| UGANDA \| 1 \| \| UKRAINE \| 1 \| \| VENEZUELA \| 1 \| \| SWEDEN \| KENYA \| 1 \| \| PERU \| 1 \| \| QATAR \| 1 \| \| SWITZERLAND \| AUSTRIA \| 1 \| \| BANGLADESH \| 1 \| \| EGYPT \| 1 \| \| LEBANON \| 1 \| \| NIGERIA \| 1 \| \| PAKISTAN \| 1 \| \| SINGAPORE \| 1 \| \| TANZANIA \| 1 \| \| TURKEY \| 1 \| \| UGANDA \| 1 \| \| UKRAINE \| 1 \| \| THAILAND \| AUSTRALIA \| 2 \| \| CONGO \| 1 \| \| ITALY \| 2 \| \| MALAYSIA \| 2 \| \| UGANDA \| 1 \| \| TURKEY \| AZERBAIJAN \| 1 \| \| DENMARK \| 1 \| \| ETHIOPIA \| 1 \| \| INDONESIA \| 2 \| \| PORTUGAL \| 1 \| \| SOUTH AFRICA \| 1 \| \| UKRAINE \| 1 \| \| UGANDA \| CONGO \| 1 \| \| LEBANON \| 1 \| \| PHILIPPINES \| 1 \| \| UKRAINE \| 1 \| \| UKRAINE \| LEBANON \| 1 \| \| UNITED KINGDOM \| AUSTRALIA \| 1 \| \| AUSTRIA \| 1 \| \| BANGLADESH \| 1 \| \| BELGIUM \| 3 \| \| BRAZIL \| 3 \| \| CANADA \| 4 \| \| CZECH REPUBLIC \| 1 \| \| ECUADOR \| 1 \| \| EGYPT \| 1 \| \| FRANCE \| 1 \| \| GREECE \| 1 \| \| HONG KONG \| 1 \| \| ICELAND \| 1 \| \| IRAN \| 3 \| \| IRELAND \| 2 \| \| ITALY \| 3 \| \| JAPAN \| 1 \| \| KENYA \| 1 \| \| LEBANON \| 1 \| \| MEXICO \| 1 \| \| NETHERLANDS \| 2 \| \| NIGERIA \| 4 \| \| NORWAY \| 1 \| \| PAKISTAN \| 2 \| \| PERU \| 1 \| \| PORTUGAL \| 1 \| \| QATAR \| 2 \| \| SAUDI ARABIA \| 1 \| \| SIERRA LEONE \| 1 \| \| SLOVAKIA \| 1 \| \| SLOVENIA \| 1 \| \| SOUTH AFRICA \| 1 \| \| SPAIN \| 2 \| \| SWEDEN \| 2 \| \| SWITZERLAND \| 2 \| \| THAILAND \| 2 \| \| TURKEY \| 1 \| \| UGANDA \| 1 \| \| UKRAINE \| 1 \| \| USA \| ARGENTINA \| 3 \| \| AUSTRALIA \| 10 \| \| AUSTRIA \| 1 \| \| AZERBAIJAN \| 1 \| \| BANGLADESH \| 1 \| \| BELGIUM \| 1 \| \| BRAZIL \| 5 \| \| CAMEROON \| 1 \| \| CANADA \| 13 \| \| CONGO \| 1 \| \| CZECH REPUBLIC \| 1 \| \| DENMARK \| 4 \| \| EGYPT \| 2 \| \| ESTONIA \| 2 \| \| FINLAND \| 1 \| \| FRANCE \| 6 \| \| GERMANY \| 19 \| \| HONDURAS \| 1 \| \| HONG KONG \| 1 \| \| HUNGARY \| 1 \| \| INDIA \| 7 \| \| IRAN \| 8 \| \| ITALY \| 8 \| \| JAPAN \| 6 \| \| KENYA \| 1 \| \| KOREA \| 10 \| \| LITHUANIA \| 1 \| \| MALAYSIA \| 2 \| \| MEXICO \| 1 \| \| MOROCCO \| 1 \| \| NETHERLANDS \| 4 \| \| NIGERIA \| 3 \| \| POLAND \| 3 \| \| PORTUGAL \| 2 \| \| QATAR \| 1 \| \| RWANDA \| 2 \| \| SAINT KITTS AND NEVIS \| 1 \| \| SAUDI ARABIA \| 1 \| \| SIERRA LEONE \| 1 \| \| SINGAPORE \| 1 \| \| SOUTH AFRICA \| 2 \| \| SPAIN \| 5 \| \| SWAZILAND \| 1 \| \| SWEDEN \| 4 \| \| SWITZERLAND \| 3 \| \| TANZANIA \| 1 \| \| THAILAND \| 5 \| \| TURKEY \| 2 \| \| UGANDA \| 1 \| \| UKRAINE \| 1 \| \| UNITED KINGDOM \| 16 \| \| VENEZUELA \| 1 \| |
