# Supplementary material for: Bibliometric analysis on CRISPR/Cas: a potential Sherlock Holmes for disease detection
Source: Front Mol Biosci. 2024 Jul 11;11:1383268. doi: 10.3389/fmolb.2024.1383268 (PMC11269658; doi:10.3389/fmolb.2024.1383268)
Supplement: Supplementary file 5 [file Table3.docx]

| **Supplementary Table 3:** Country-wise citation score on CRISPR-based disease detection |
| --- |
| \| **Country** \| **TC** \| **Average Article Citations per Year** \| \| --- \| --- \| --- \| \| USA \| 15467 \| 70.00 \| \| CHINA \| 10573 \| 16.70 \| \| INDIA \| 1339 \| 16.90 \| \| GERMANY \| 1054 \| 32.90 \| \| UNITED KINGDOM \| 1041 \| 43.40 \| \| KOREA \| 857 \| 22.00 \| \| CANADA \| 726 \| 36.30 \| \| IRAN \| 712 \| 21.60 \| \| GEORGIA \| 644 \| 161.00 \| \| SAUDI ARABIA \| 605 \| 43.20 \| \| JAPAN \| 579 \| 32.20 \| \| THAILAND \| 543 \| 36.20 \| \| MALAYSIA \| 384 \| 34.90 \| \| SWITZERLAND \| 383 \| 47.90 \| \| AUSTRALIA \| 372 \| 31.00 \| \| ITALY \| 327 \| 23.40 \| \| CZECH REPUBLIC \| 275 \| 45.80 \| \| QATAR \| 272 \| 90.70 \| \| SINGAPORE \| 261 \| 37.30 \| \| NETHERLANDS \| 234 \| 23.40 \| \| SPAIN \| 233 \| 12.90 \| \| FRANCE \| 202 \| 13.50 \| \| BRAZIL \| 201 \| 18.30 \| \| SWEDEN \| 190 \| 31.70 \| \| LITHUANIA \| 141 \| 47.00 \| \| POLAND \| 121 \| 24.20 \| \| TURKEY \| 111 \| 12.30 \| \| SUDAN \| 100 \| 100.00 \| \| KAZAKHSTAN \| 82 \| 41.00 \| \| BANGLADESH \| 70 \| 23.30 \| \| INDONESIA \| 49 \| 16.30 \| \| SOUTH AFRICA \| 47 \| 23.50 \| \| MEXICO \| 45 \| 11.20 \| \| YEMEN \| 42 \| 42.00 \| \| BELGIUM \| 33 \| 8.20 \| \| UNITED ARAB EMIRATES \| 30 \| 15.00 \| \| AUSTRIA \| 29 \| 7.20 \| \| NIGERIA \| 28 \| 14.00 \| \| IRELAND \| 25 \| 12.50 \| \| PAKISTAN \| 23 \| 2.60 \| \| PORTUGAL \| 23 \| 7.70 \| \| HONG KONG \| 21 \| 3.50 \| \| PERU \| 20 \| 5.00 \| \| ARGENTINA \| 19 \| 6.30 \| \| MOROCCO \| 19 \| 19.00 \| \| GREECE \| 17 \| 17.00 \| \| ROMANIA \| 17 \| 8.50 \| \| IRAQ \| 16 \| 16.00 \| \| ETHIOPIA \| 15 \| 15.00 \| \| SLOVAKIA \| 14 \| 14.00 \| \| EGYPT \| 12 \| 12.00 \| \| ECUADOR \| 11 \| 11.00 \| \| OMAN \| 10 \| 10.00 \| \| DENMARK \| 9 \| 4.50 \| \| NEW ZEALAND \| 9 \| 9.00 \| \| CHILE \| 8 \| 8.00 \| \| VIETNAM \| 7 \| 7.00 \| \| MALI \| 4 \| 4.00 \| \| TANZANIA \| 4 \| 4.00 \| \| KENYA \| 3 \| 1.50 \| \| FAROE \| 2 \| 2.00 \| \| ESTONIA \| 0 \| 0.00 \| \| NEPAL \| 0 \| 0.00 \| |
