# Supplementary material for: Bibliometric analysis on CRISPR/Cas: a potential Sherlock Holmes for disease detection
Source: Front Mol Biosci. 2024 Jul 11;11:1383268. doi: 10.3389/fmolb.2024.1383268 (PMC11269658; doi:10.3389/fmolb.2024.1383268)
Supplement: Supplementary file 6 [file Image1.pdf]

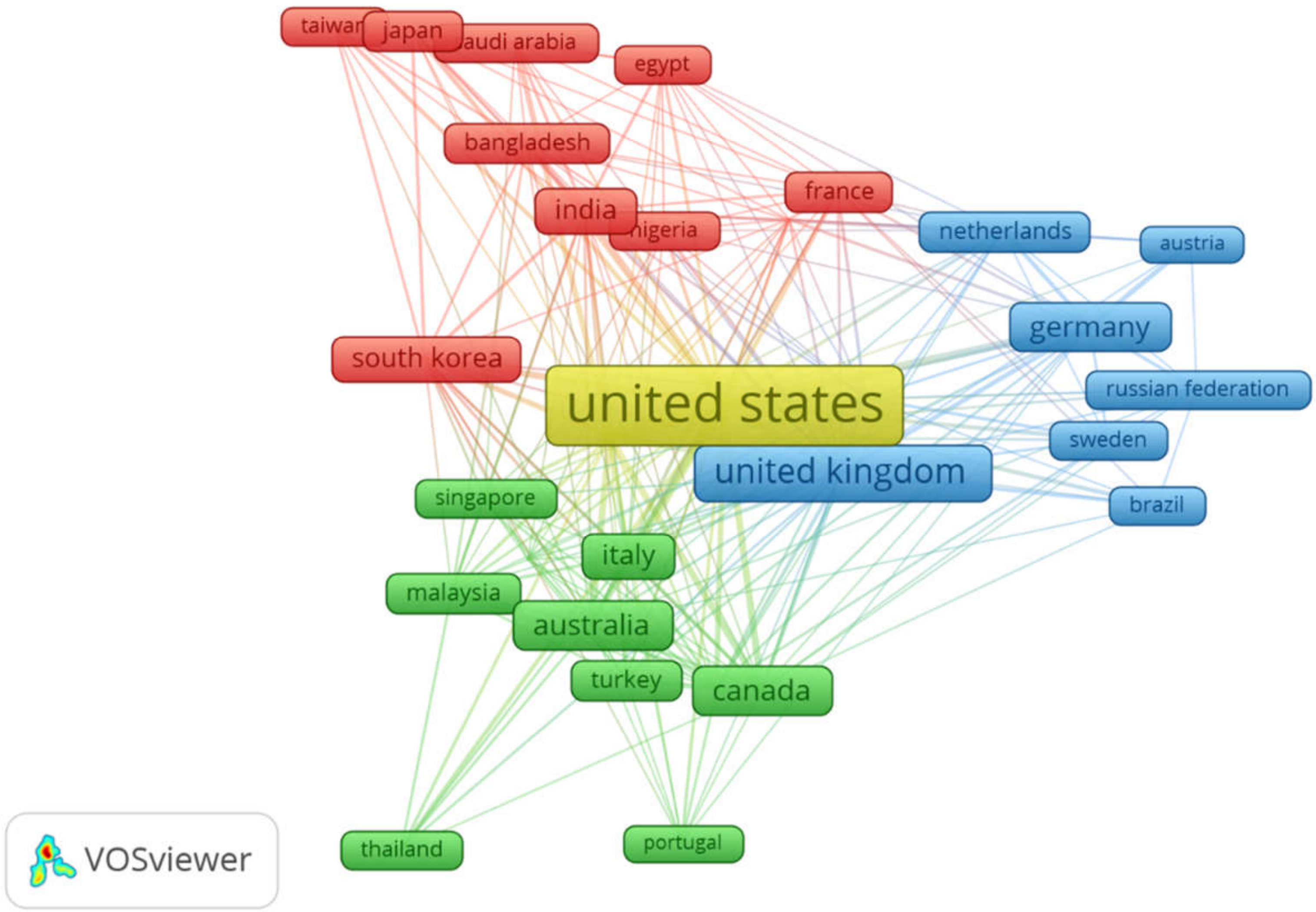

Fig S1: A network has been used to visually illustrate the collaborative study of CRISPR-based disease detection conducted across multiple countries. Only countries that have a minimum of five published works were considered. The graphic displays clusters, which are groups of closely interconnected nodes or items, and each cluster is represented by a distinct color. Each of individual item is exclusively assigned to a single cluster, and its color indicates its membership inside that cluster. Connections between objects are depicted by lines, while the proximity of two elements indicates the extent of their correlation.
